# Identification of a New Mutation p.P88L in Connexin 50 Associated with Dominant Congenital Cataract

**DOI:** 10.3389/fcell.2022.794837

**Published:** 2022-04-21

**Authors:** Aixia Jin, Qingqing Zhao, Shuting Liu, Zi-bing Jin, Shuyan Li, Mengqing Xiang, Mingbing Zeng, Kangxin Jin

**Affiliations:** ^1^ State Key Laboratory of Ophthalmology, Zhongshan Ophthalmic Center, Sun Yat-Sen University, Guangdong Provincial Key Laboratory of Ophthalmology and Visual Science, Guangzhou, China; ^2^ Beijing Institute of Ophthalmology, Beijing Tongren Eye Center, Beijing Tongren Hospital, Capital Medical University, Beijing Ophthalmology and Visual Science Key Laboratory, Beijing, China; ^3^ Department of Biochemistry and Biophysics, Peking University Health Science Center, Beijing, China; ^4^ Hainan Eye Hospital, Hainan Key Laboratory of Ophthalmology, Zhongshan Ophthalmic Center, Sun Yat-sen University, Haikou, China

**Keywords:** congenital cataract, genetic mutation, exome sequencing, connexin, gap junction, hemichannel

## Abstract

Congenital hereditary cataract is genetically heterogeneous and the leading cause of visual impairment in children. Identification of hereditary causes is critical to genetic counselling and family planning. Here, we examined a four-generation Chinese pedigree with congenital dominant cataract and identified a new mutation in *GJA8* via targeted exome sequencing. A heterozygous missense mutation c.263C > T, leading to a proline-to-Leucine conversion at the conserved residue 88 in the second transmembrane domain of human connexin 50 (Cx50), was identified in all patients but not in unaffected family members. Functional analyses of the mutation revealed that it disrupted the stability of Cx50 and had a deleterious effect on protein function. Indeed, the mutation compromised normal membrane permeability and gating of ions, and impeded cell migration when overexpressed. Together, our results expand the pathogenic mutation spectrum of Cx50 underlying congenital cataract and lend more support to clinical diagnosis and genetic counseling.

## Introduction

The mammalian lens is a transparent organ whose main function is to transmit and focus light onto the retina. During development, the lens is originated from invaginated surface ectoderm that reciprocally induces primordium optic cup (Cvekl and Zhang, 2017). Three cell types, the lens epithelial cells (LECs) and the differentiating and mature fiber cells, compose the lens. The LECs form a monolayer covering the anterior surface and the fiber cells build the bulk of the organ. Fiber cells are differentiated from LECs through a process involving cell proliferation, migration and elongation, and loss of nuclei and cytoplasmic organelles (Cvekl and Ashery-Padan, 2014; Cvekl and Zhang, 2017).

Lens growth occurs almost throughout the lifetime, though extremely slow in adulthood. Genetic or environmental factors interfering with the process could lead to congenital or developmental cataracts, characterized by cloudiness and opacity of the lens. For genetic cataracts, nearly half of them are caused by mutations in the main structural protein crystallins, and surprisingly, about a quarter by abnormalities in gap junction protein connexins (Shiels et al., 2010). With the advances of next generation sequencing technologies, more and more new genetic mutations are identified. For example, some new mutations in Cx46, Cx50 and other genes were recently found to be associated with cataract and other visual impairments (Berry et al., 2020; Fan et al., 2020). Despite all the mutations identified thus far, the known mutations can explain only a small fraction of hereditary diseases.

Three major connexins, Cx50, Cx46 and Cx43, are dynamically expressed in mammalian lens cells (Bassnett and Šikić, 2017; Cvekl and Zhang, 2017; Donaldson et al., 2017). In LECs, connexins are predominantly Cx50 and Cx43, and include some Cx46. Cx43 is diminishing in the differentiating fiber cells and almost disappears from mature fiber cells. On the contrary, Cx46 gradually increases in the differentiating and mature fiber cells (Mathias et al., 2010; Berthoud and Ngezahayo, 2017). Though the structure and function of the three connexins are similar, there is only partial redundancy in their function. For example, the phenotype of smaller lens in *Cx50* knockout mice could not be rescued by replacement with *Cx46* knockin (Sellitto et al., 2004). The nature of the dynamic and differential expression patterns of the connexins is still not understood.

Recently, near-atomic level structure of sheep lens Cx46/50 has been resolved with electron cryo-microscopy (CryoEM) (Myers et al., 2018; Flores et al., 2020), providing us clues to how the architectural stability, structure and function of gap junction communication channels are maintained. Given that mammalian connexins are highly conserved, especially in functional domains, the native structural details are very helpful for understanding how human Cx50 works *in vivo*. Despite this, the importance of individual amino acid residue and its contribution to protein structure and function have largely been gained from patients and causative mutational studies. In this study, we identified a new mutation c.263C > T in *GJA8* gene, corresponding to p.P88L in Cx50, from a four-generation pedigree with dominant congenital cataract in Hainan province of China. This finding adds a new mutation to the mutation spectrum and expands our understanding of the structural base and function of human Cx50.

## Materials and Methods

### Patients and Deoxyribonucleic Acid Collection

A four-generation Chinese family with hereditary dominant congenital cataract was recruited at Hainan Eye Hospital, Zhongshan Ophthalmic Center. We obtained the medical histories of 24 family members, among which 11 members were available in this study, including 9 affected and 2 unaffected individuals. The diagnosis of cataract was confirmed by a series of ophthalmologic examinations. Genomic DNA was extracted from peripheral blood cells using the QIAamp DNA Blood Mini Kit (Qiagen, China).

The medical protocol was approved by the Committee of Human Ethics in Medical Research in Zhongshan Ophthalmic Center and consistent with the Declaration of Helsinki. The written informed consent was obtained from each participant or their legal custodians.

### Targeted Exome Sequencing

A minimum of 3 ug DNA was used for the indexed Illumina libraries according to manufacturer’s protocol (MyGenostics Inc., Beijing, China). DNA fragments with sizes ranging from 350 to 450 bp and those including the adapter sequences were selected for the DNA libraries. Next, targeted exome sequencing was selected by a gene capture strategy, using the GenCap Exome Enrichment Kit V4.0 (MyGenostics Inc., Beijing, China). The biotinylated capture probes (80–120-mer) were designed to tile the targeted exons with non-repeated regions. The kit was designed to detect exome of 3,086 genes whose mutations are known to be pathogenic. The captured DNA was amplified and purified with SPRI beads (Beckman Coulter, Brea, CA, United States). The enriched libraries were sequenced with an Illumina NextSeq 500 sequencer for paired-end reads of 150 bp with an average depth of 120X. The data was deposited to GSA-Human with accession number HRA001484 (https://bigd.big.ac.cn/gsa-human/browse/HRA001484).

### Mutation and Bioinformatics Analysis

Following sequencing, raw image files were processed using Bcl2Fastq software (Bcl2Fastq 2.18.0.12, Illumina, Inc.) for base calling and raw data generation. Low-quality variations were filtered out using a quality score ≥20. Short Oligonucleotide Analysis Package (SOAP) aligner software (SOAP2.21; soap.genomics.org.cn/soapsnp.html) was then used to align the clean reads to the reference human genome (hg19). Polymerase chain reaction (PCR) duplicates were removed using the Picard program. Subsequently, single nucleotide polymorphisms (SNPs) were determined using the SOAPsnp program, reads were realigned using Burrows-Wheeler Aligner software 0.7.15, and the deletions and insertions (InDels) were detected using Genome Analysis Toolkit software 3.7. The identified SNPs and InDels were annotated using the Exome-assistant program (http://122.228.158.106/exomeassistant). The MagicViewer was used to view the short-read alignment, and confirm the candidate SNPs and InDels.

To determine pathogenicity, non-synonymous variants were evaluated using the following programs: PolyPhen2 (http://genetics.bwh.harvard.edu/pph2/), PROVEAN (Protein Variation Effect Analyzer V1.1.3, http://provean.jcvi.org/), PANTHER (Protein Analysis Through Evolutionary Relationships, www.pantherdb.org), SDM2 (Site Directed Mutator, http://marid.bioc.cam.ac.uk/sdm2/), PMut (http://mmb.irbbarcelona.org/PMut) and MutationTaster (http://doro.charite.de/MutationTaster/index.html). The calculation of hydrophobicity score was determined by ProtScale (https://web.expasy.org/protscale/), and image was redrawn with GraphPad Prism (GraphPad Software, La Jolla, CA). The prediction of mutational protein structure was obtained from RoseTTAFold (Baek et al., 2021) (https://robetta.bakerlab.org/), a fast and accurate deep learning-based modeling method comparable to AlphaFold2. Wild type and mutational protein structures and amino acid interactions were captured with Pymol Molecular Graphics System (v2.6.0a0).

### Sanger Sequencing

All mutations identified by the Illumina NextSeq 500 sequencer were confirmed by Sanger sequencing. To validate the mutation in *GJA8* gene, a 262bp PCR product was amplified from each patient genome DNA. The following PCR primers were used: 5′- ATGAGCAATCCGACTTCG -3’ (forward), and 5′- TTC​TTA​GTG​CCT​TTG​CTG​C -3’ (reverse). High fidelity PrimeSTAR Max DNA Polymerase (Takara, Beijing, China) was used to avoid amplification errors. The amplified DNA fragments were purified and sequenced with an ABI PRISM 3730 genetic analyzer (Applied Biosystems; Thermo Fisher Scientific, Inc.). The sequence reading was carefully examined and confirmed manually.

### Plasmid Construction and Cell Scratch Wound Analysis

The human *GJA8* open reading frame (ORF) sequence (NM_005,267.5) and ORF with the point mutation (*P88L*, c.263C > T) were cloned into the pCIG plasmid, a mammalian expression vector containing a sequence of the CMV enhancer, chicken *ß*-actin promoter, multiple cloning sites (MCS), internal ribosome entry site (IRES), *eGFP,* and rabbit *ß*-globin polyA sequences, as described previously (Jin et al., 2015).

The human LECs were purchased from ATCC (Cat# CRL-11421)). Cells were cultured in 10 cm dishes following the manufacture’s protocol. They were then transfected with 40 µg of each plasmid using Hieff Trans™ Liposomal Transfection Reagent (Yeasen, Cat# 40802ES03). After reaching a confluent monolayer, cells were scraped with a 10 µL pipette tip to create scratches of similar sizes. Images were captured from the marked region every 12 h (hours) and were analyzed quantitatively with the software ImageJ. The wound closure ratio was calculated as one minus the ratio of the blank area at each timepoint with the initial blank area (1 - blank area/initial blank area). The statistical analyses and line charts were processed and generated with Graphpad Prism. The non-parametric method Friedman test was used to determine the statistical difference and a value of *p* < 0.05 is considered to be significantly different.

### EdU Labeling

Human LECs transfected with 3 µg of pCIG-vector, -hGJA8 or -P88L were cultured in 6-well plate for 48 h. Then 1 × 10^5^ cells were plated in each well (12-well plate) containing a glass coverslip pre-coated with poly-l-Lysine and laminin. One day following plating, EdU (5-ethynyl-2′-deoxyuridine) was added into the culture medium to a final concentration of 10 μM, and cells were cultivated for 24 h or 48 h. They were fixed with 4% formaldehyde for 15 min, washed with PBS and blocked with 10% donkey serum in PBS for 1h under room temperature. GFP antibody (1:1,000 dilution, Abcam, cat# ab6673) was added and incubated at 4 °C overnight. Cells were washed with PBS and incubated with secondary antibodies conjugated with Alexa-488 for 1 h. EdU staining was carried out according to the manufacturer’s instruction (Click-iT EdU Cell Proliferation Kit, Thermofisher Scientific, Cat# C10338). Images were captured with Zeiss confocal 700 system. GFP^+^, EdU^+^, and GFP^+^Edu^+^ double-positive cells were counted and analyzed.

### Patch Clamp Analysis of Membranal Voltage Gating and Ion Permeability

Human LECs were transfected with pCIG-vector, -*GJA8*, or -*P88L*. To obtain the whole-cell recording, GFP-positive cells were bathed in external solution which contained 135 mM NaCl, 5 mM KCl, 2 mM CsCl, 2 mM CaCl2, 1 mM MgCl2, 5 mM HEPES, 5 mM dextrose, 2 mM pyruvate, and 1 mM BaCl2, pH 7.4. The patch pipette solution (internal solution) contained 125 mM CsCl, 10 mM EGTA, 0.5 mM CaCl2, and 10 mM HEPES and its pH value was adjusted to 7.2. Recordings were performed with the Axon 700B amplifier and Clampfit software (v11). The glass patch pipette had a resistance of 3–5 MΩ in bath solution. Data were sampled at minimal start intervals and low-pass was filtered at 1 kHz. Leak subtraction was not employed.

### Antibodies and Cell Immunofluorescence

Human LECs transfected with pCIG-vector, -hGJA8 or -P88L were cultured for 72 h and fixed in 4% paraformaldehyde in PBS (pH7.4) for 5 min. They were then washed with 1x PBS 3 times and blocked with 5% normal sheep serum in PBS for 1 h. Primary antibodies against Cx50 (1:200 dilution, Santa Cruz, cat# sc-3738,011) and GFP (1:500 dilution, Abcam, cat# ab6673) were added and incubated at 4°C overnight. Cells were washed with PBS 3 times to remove free primary antibodies and secondary antibodies conjugated with Alexa-488 or Alexa-594 were added and incubated for 1 h at room temperature. Extra antibodies were washed away with PBS. Images were captured with Zeiss confocal 700 system.

## Results

### Clinical Data and Findings

**FIGURE 1:**
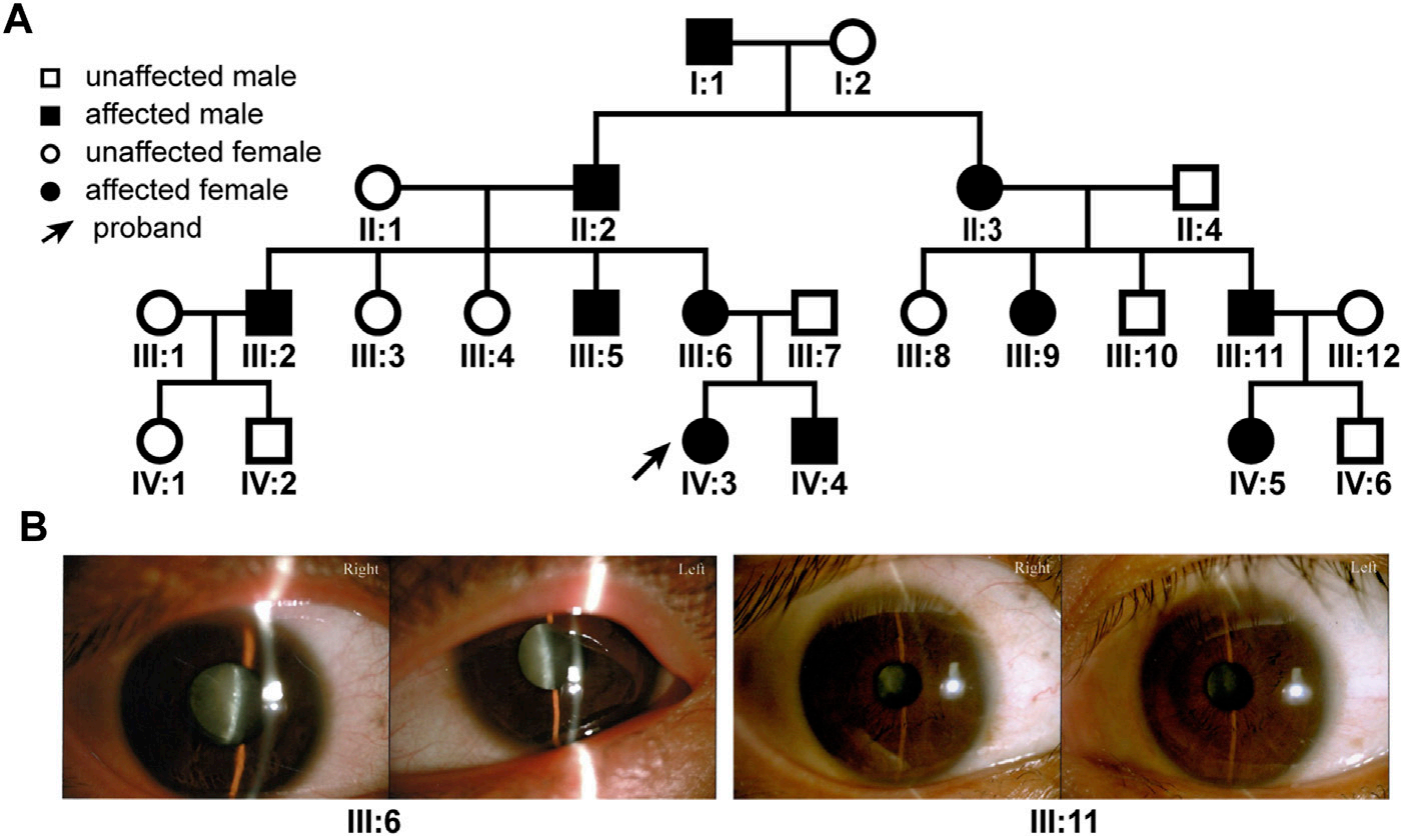
The family tree of the patient pedigree and cataract phenotype. **(A)** The four-generation 24-member pedigree with dominant congenital cataract. **(B)** Varied cataract types and severity in patients. Photographs of eyes of two patients were shown. Patient III:6 has bilateral total cataracts. Patient III: 11 has a nuclear cataract in the right eye and a zonular cataract in the left eye.

Due to the island and mountainous landscape, Hainan province has relatively more undiagnosed families with hereditary diseases. We recently found a four-generation pedigree with congenital dominant cataract in the province (Figure 1A). There were a total of 11 affected and 13 unaffected members in the pedigree. Eight members (II:3, III:5, 6, 9, 11, IV:3, 4, 5) had sought treatment in our hospital, all with bilateral cataract (Figure 1B), 4 with bilateral nystagmus (III:5, 6, 11, IV:3), 3 with bilateral or unilateral strabismus (III:6, 9, 11). The nystagmus and strabismus were secondary diseases following cataract. The category of cataract types and severity varied among patients (Figure 1B); however, upon inquiry of patients and family members, all affected members in the pedigree were confirmed to have congenital bilateral cataracts.

### Mutation Identification and Analyses

To identify the causative mutation(s), we sent 3 patient genomic DNA samples (II:3, III:11, IV:5) for targeted exome sequencing, which was designed to specifically focus on the exome of a panel of 3,086 genes known to be pathogenic when mutated. A total of 8 mutations, c.2010 + 10G > A (splicing) in *ACTN4*, c.1697A > G (p.H566R) in *ATP2A2*, c.242C > T (p.P81L) in *CFHR5*, c.263C > T (p.P88L) in *GJA8*, c.4475A > C (p.Q1492P) in *MED13*, c.1021G > T (p.G341C) in *PRX*, c.3634C > T (p.P1212S) in *SHANK3*, and c.697G > C (p.V233L) in *SLC26A4*, were present in all 3 patients. Except for the *GJA8* mutation, the other 7 gene mutations were autosomal-recessive or predicted to be benign or uncertain to be pathogenic, and more importantly, no correlated diseases or symptoms were previously diagnosed in patients with these mutations. The c.263C > T mutation in *GJA8* was verified in all 3 patients by Sanger sequencing. We further examined genomic DNA from the other 8 inpatient and outpatient individuals (I:1, III:5, 6, 9, 12, IV:3, 4, 6) in the pedigree by Sanger sequencing. The c.263C > T mutation was co-segregated with all affected but not with unaffected individuals.

**FIGURE 2:**
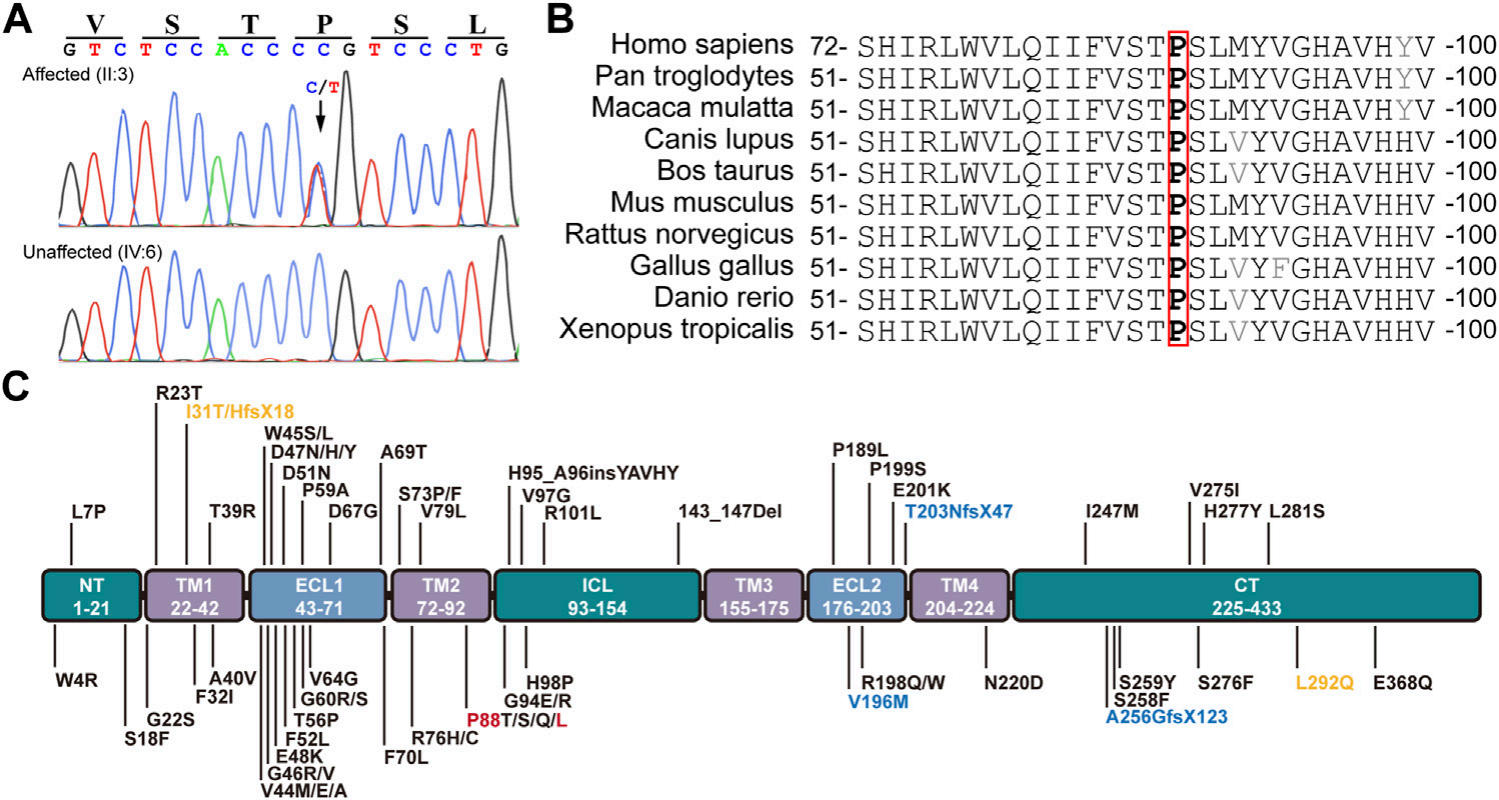
Mutation identification and analyses. **(A)** The c.262C > T heterozygous mutation in GJA8 was confirmed in all affected but not in unaffected family members by the Sanger sequencing. This mutation results in a p.P88L amino acid change in the second transmembrane domain in Cx50. **(B)** The P88 (highlighted in red box) and neighbor amino acid residues were conserved in the indicated vertebrates. **(C)** Known pathogenic mutations in human Cx50 are listed according to their positions in the functional domains. Dominant mutations are listed in black, recessive mutations in blue, and uncertain mutations in orange. Letters in red denote the mutation in this study. NT (N-terminus), ICL (intracellular loop), and CT (C-terminus) are intracellular regions (in green boxes). TM1-4 indicate the four transmembrane regions (in purple boxes). ECL1-2 are extracellular loops (in light blue boxes).

The conversion of c.263C > T leads to the substitution of Leucine (L) for Proline (P) at the 88th residue (p.P88L) in the 2nd transmembrane domain of Cx50 (Figure 2A). The P88 residue is extremely conserved in all examined homologues from the *Xenopus*, zebrafish, chicken, rat, mouse, cow, dog, mulatta, chimpanzee, and human (Figure 2B), as well as in counterparts of other connexins (Arora et al., 2006; Ge et al., 2014; Myers et al., 2018), indicating an indispensable role for it in maintaining connexin structure and function (Shiels et al., 1998; Arora et al., 2006; Vanita et al., 2008a; Ge et al., 2014). The mutation is an unreported mutation (Figure 2C and Table 1). It is not present in databases including Ensembl, 1,000 Genomes, dbSNP, HapMap, ClinVar, or gnomAD.

**TABLE 1:** Reported human GJA8 mutations and related diseases.

Nucleotide Change	Amino Acid Change	Mutation Type	Hetero-/Homozygous	AR or AD	Related Diseases	Reference
c.10T > A	p.W4R	missense	Het	AD	congenital cataract	Zhang et al. (2018a)
c.20T > C	p.L7P	missense	Het	AD	congenital cataract	Mackay et al. (2014)
c.53C > T	p.S18F	missense	Het	AD	congenital cataract	Micheal et al. (2018)
c.64G > A	p.G22S	missense	Het	AD	congenital pulverulent cataract	Ye et al. (2019)
c.68G > C	p.R23T	missense	Het	AD	congenital nuclear cataract	Willoughby et al. (2003)
c.89dupT	p.I31HfsX18	Insertion, frameshift	*Homo*	AR (AD?)	congenital cataract	Ma et al. (2016)
c.92T > C	p.I31T	missense	Het	AD	congenital nuclear cataract	Wang et al. (2009)
c.94T > C	p.F32I	missense	Het	AD	congenital cataract	Dang et al. (2016)
c.116C > G	p.T39R	missense	Het	AD	congenital cataract with microcornea	Sun et al. (2011); Ceroni et al. (2019)
c.119C > T	p.A40V	missense	Het	AD	congenital cataract	Ma et al. (2016)
c.130G > A	p.V44M	missense	Het	AD	congenital cataract	Mohebi et al. (2017); Zhang et al. (2018b)
c.131T > A	p.V44E	missense	Het	AD	congenital cataract with microcornea	Devi and Vijayalakshmi, (2006)
c.131T > C	p.V44A	missense	Het	AD	congenital nuclear cataract	Zhu et al. (2014)
c.134G > C	p.W45S	missense	Het	AD	congenital cataract with microcornea	Vanita et al. (2008b); Ma et al. (2016)
c.134G > T	p.W45L	missense	Het	AD	congenital cataract, w/o microcornea	Mohebi et al. (2017); Ceroni et al. (2019)
c.136G > A	p.G46R	missense	Het	AD	congenital cataract with microcornea	Sun et al. (2011); Fan et al. (2020)
c.137G > T	p.G46V	missense	Het	AD	congenital cataract	Minogue et al. (2009)
c.139G > A	p.D47N	missense	Het	AD	congenital nuclear/zonular/pulverulent cataract	Arora et al. (2008); He et al. (2011); Wang et al. (2011); Liang et al. (2015); Shen et al. (2017); Gunda et al. (2018)
c.139G > C	p.D47H	missense	Het	AD	congenital nuclear/zonular/pulverulent cataract	Li et al. (2013)
c.139G > T	p.D47Y	missense	Het	AD	congenital cataract	Lin et al. (2008)
c.142G > A	p.E48K	missense	Het	AD	Congenital zonular/nuclear/pulverulent cataract	Berry et al. (1999)
c.151G > A	p.D51N	missense	Het	AD	congenital cataract, sclerocornea, microphthalmia	Ma et al. (2016); Ceroni et al. (2019)
c.154T > C	p.F52 L	missense	Het	AD	congenital cataract	Li et al. (2019a)
c.166A > C	p.T56P	missense	Het	AD	congenital nuclear cataract	Hadrami et al. (2019)
c.175C > G	p.P59A	missense	Het	AD	congenital cataract	Yu et al. (2016); Micheal et al. (2018)
c.178G > C	p.G60R	missense	Het	AD	congenital zonular cataract	Berry et al. (2020)
c.178G > A	p.G60S	missense	Het	AD	congenital cataract	Ding et al. (2020)
c.191T > G	p.V64G	missense	Het	AD	congenital cataract	Zheng et al. (2005)
c.200A > G	p.D67G	missense	Het	AD	congenital cataract	Reis et al. (2013)
c.205G > A	p.A69T	missense	Het	AD	congenital cataract	Li et al. (2020)
c.208T > C	p.F70L	missense	Het	AD	congenital cataract with microphthalmia	Ceroni et al. (2019)
c.217T > C	p.S73P	missense	Het	AD	congenital cataract	Li et al. (2019b)
c.218C > T	p.S73F	missense	Het	AD	congenital nuclear cataract	Yang et al. (2015)
c.226C > T	p.R76C	missense	Het	AD	congenital cataract	Reis et al. (2013)
c.227G > A	p.R76H	missense	Het	AD	congenital cataract	Yu et al. (2016); Wang et al. (2020)
c.235G > C	p.V79L	missense	Het	AD	congenital cataract	Vanita et al. (2006)
c.262C > A^#1^	p.P88T	missense	Het	AD	congenital cataract	Ge et al. (2014)
c.262C > T	p.P88S	missense	Het	AD	congenital zonular/pulverulent cataract	Shiels et al. (1998); Berry et al. (2020)
c.263C > A^#2^	p.P88Q	missense	Het	AD	congenital cataract	Arora et al. (2006); Vanita et al. (2008a)
c.263C > T	p.P88L	missense	Het	AD	congenital cataract	this study
c.280G > C	p.G94R	missense	Het	AD	congenital cataract	Ma et al. (2018)
c.280G > A	p.G94R	missense	Het^#9^	AD^#9^	no lens, microphthalmia, coloboma, etc.	Ceroni et al. (2019)
c.281G > A	p.G94E	missense	Het	AD	congenital cataract, sclerocornea	Ma et al. (2018)
c.285ins	p.H95_A96insYAVHY	insertion	Het	AD	congenital cataract	Cui et al. (2018)
c.290T > G	p.V97G	missense	Het^#10^	AD^#10^	congenital cataract, microphthalmia, glaucoma, etc.	Ceroni et al. (2019)
c.293A > C	p.H98P	missense	Het	AD	congenital cataract	Mackay et al. (2014)
c.301G > T	p.R101L	missense	Het	AD	congenital cataract	Mohebi et al. (2017)
c.426_440del	p.143_147del	deletion	Het	AD	congenital cataract	Min et al. (2016)
c.433G > T	p.G145W	missense	Het	AD	congenital cataract	Ren et al. (2017)
c.565C > T	p.P189L	missense	Het	AD	congenital cataract with microcornea	Hansen et al. (2007)
c.586G > A^#3^	p.V196M	missense	*Homo*	AR	congenital cataract	Ponnam et al. (2013)
c.592C > T	p.R198W	missense	Het	AD	congenital cataract with microcornea	Hu et al. (2010)
c.593G > A	p.R198Q	missense	Het	AD	developmental cataract	Devi and Vijayalakshmi, (2006)
c.595C > T^#4^	p.P199S	missense	Het	AD	congenital cataract	Ponnam et al. (2013)
c.601G > A	p.E201K	missense	Het	AD	congenital perinuclear cataract	Su et al. (2013)
c.607insA^#5^	p.T203NfsX47	insertion, frameshift	*Homo*	AR	congenital cataract	Ponnam et al. (2007)
c.658A > G	p.N220D	missense	Het	AD	congenital cataract (or unaffected)	Kuo et al. (2017)
c.741T > G	p.I247M	missense	Het	AD	congenital zonular pulverulent cataract	Polyakov et al. (2001); Reis et al. (2013) ^#12^
c.766insG^#6^	p.A256GfsX123^#11^	insertion, frameshift	*Homo*	AR	congenital cataract	Schmidt et al. (2008)
c.773C > T	p.S258F	missense	Het	AD	congenital nuclear cataract	Gao et al. (2010)
c.776C > A^#7^	p.S259Y	missense	Het	AD	congenital cataract	Hansen et al. (2009)
c.823G > A	p.V275I	missense	Het	AD	developmental cataract	Zhou et al. (2011)
c.827C > T	p.S276F	missense	Het	AD	congenital nuclear pulverulent cataract	Yan et al. (2008)
c.829C > T	p.H277Y	missense	Het	AD	congenital nuclear pulverulent cataract	Chen et al. (2015)
c.842T > C^#8^	p.L281S^#8^	missense	Het	AD	congenital zonular cataract	Kumar et al. (2011)
c.875T > A	p.L292Q	missense	n/a	n/a	congenital cataract, coloboma, etc.	Ceroni et al. (2019)
c.1102G > C	p.E368Q	missense	Het	AD	congenital cataract	Senthil Kumar et al. (2016)

Notes, #1–8: They were listed as c.264C > A, c.262C > A, c.649G > A, c.658C > T, c,670insA, c.776insG, c.836C > A, c.905T > C (p.L281C) in the original publications, respectively, but were re-numbered with the common standard (39). #9–10: The mutations were justified as heterozygous and dominant according to the Sanger sequencing results in the original publication. #11: The amino acid changes were not described in the original publication. Here it shows the inferred sequence from nucleotide changes. #12: It is present in 0.7% of the European American population and is most likely a polymorphism.

### P88L Mutation Disrupts Cx50 Structure and Function

**FIGURE 3:**
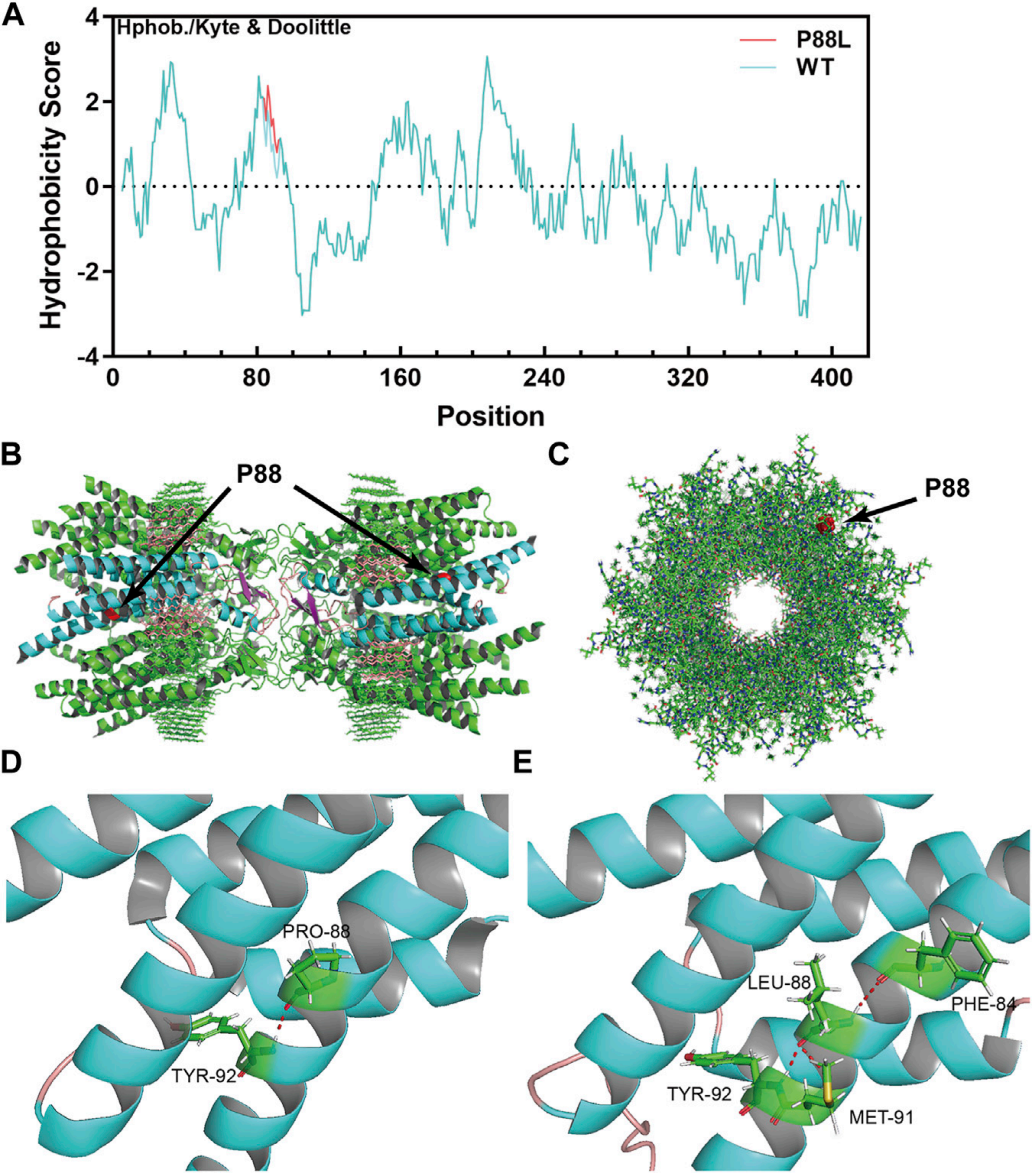
The P88L mutation increases local hydrophobicity and transforms 3-dimentional conformation of Cx50. **(A)** The hydrophobicity score was calculated with the Kphob/Kyle & Doolittle method on ProtScale. It is obvious that the local hydrophobicity score is increased around position 88 in the P88L mutant. **(B)** The horizontal view of the two P88 positions (labeled in red) in the gap junction channel made of a dodecamer of sheep Cx50. **(C)** The vertical view of one P88 position (labeled in red) in the gap junction channel made of a dodecamer of sheep Cx50. **(D, E)** Even though the Pro88-to-Leu88 substitute keeps a hydrogen bond (in red) interaction with Tyr92, Leu88 establishes extra hydrogen bonds with Met91 and Phe84, which drastically transforms the three-dimensional conformation as shown. The structures in **(B–D)** were generated based on the protein structure file 7jjp.pdb downloaded from PDB. The mutant structure in E was predicted with RoseTTAFold. The pictures in **(B–E)** were visualized and captured with Pymol (V2.6.0a0).

To evaluate the damaging effects of P88L mutation in Cx50, we used well-endorsed programs for *in silico* predictions. PolyPhen2, PANTHER, PMut, SDM2, PROVEAN, and MutationTaster output the results of ‘Probably damaging’, ‘Probably damaging’, ‘Disease’, ‘Increased stability’, ‘Deleterious’ and ‘Disease causing’, respectively (Table 2). These results are in agreement with the increased hydrophobicity score around the mutation site (Figure 3A).

**TABLE 2:** In silico predictions of functional effects for Cx50-P88L mutation.

	PolyPhen2	PANTHER	PMut	SDM2	PROVEAN	MutationTaster
**Score**	1.000	Preservation time: 797 million years	0.93	ΔΔG = 1.56	−9.963	Probability = 0.999
						Score = 98
**Result**	Probably damaging	Probably damaging	Disease	Increased stability	Deleterious	Disease causing

The native channel structure of the 12 subunits of Cx50 and/or Cx46 has recently been elucidated with cryo-EM (Flores et al., 2020; Myers et al., 2018). P88 sits at the homo/heteromeric interface but not homo/heterotypic interface (Figures 3B,C) (Myers et al., 2018), and therefore the P88L mutation likely interrupts the Cx50 conformation or homo/heteromeric interface interactions. In the wildtype Cx50 protein, P88 maintains the transmembrane α-helix structure by establishing a hydrogen bond with Y92 (Tyr92, Figure 3D). In the mutant, even though L88 reestablishes a hydrogen bond with Y92, it also interacts with F84 and M91 through hydrogen bonds, resulting in regional and global protein conformational changes (Figure 3E). The changes might directly or indirectly disrupt the physiological function of the channels or hemi-channels.

### P88L Mutation Impedes the Migration but Not Proliferation of Lens Epithelial Cells

**FIGURE 4:**
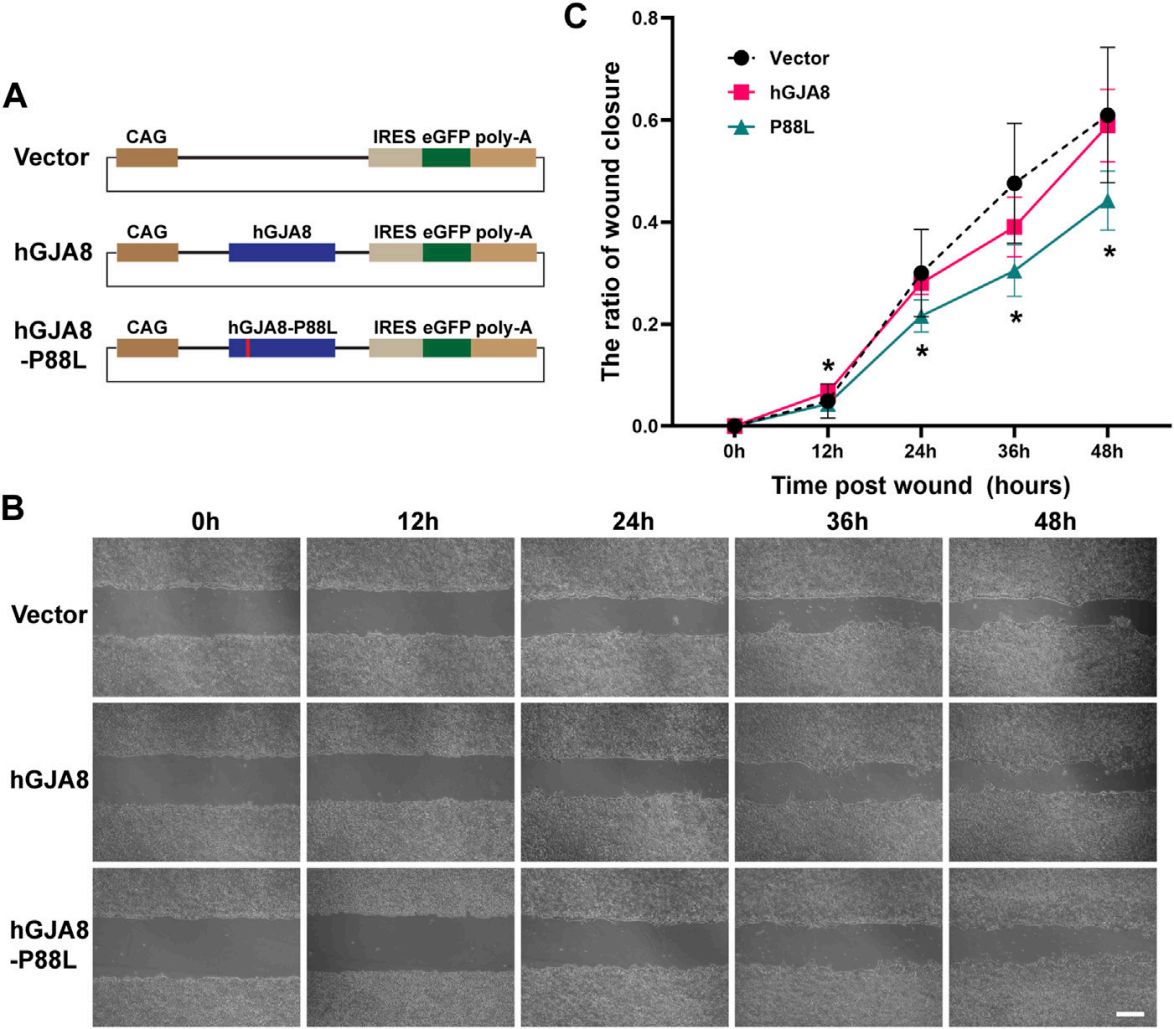
Overexpression of P88L inhibits human LECs migration. **(A)** Illustration of pCIG-vector, pCIG-hGJA8 and pCIG-hGJA8-P88L plasmids used for transfection. **(B)** Human LECs were transfected with the indicated plasmids. Cells were scraped to create wounds of similar sizes. Representative images were captured at timepoints 0 h (hour), 12, 24, 36 and 48 h after cell scratches. Scale bar: 200 µm. **(C)** The wound closure ratios were calculated at each timepoint by ImageJ. The asterisk ‘*’ represents a significant difference (*p* < 0.05; *N* = 6 for GJA8 and vector, *N* = 8 for P88L) between P88L and wildtype GJA8 or vector at the timepoint.

It has been shown that connexins including Cx50 could directly or indirectly affect cell proliferation and migration (Sellitto et al., 2004; White et al., 2007; Machtaler et al., 2014; Polusani et al., 2016; Tjahjono et al., 2020). To examine if P88L mutation could interfere with LECs proliferation and/or migration, we cloned the human wildtype *GJA8* and *P88L* into a plasmid vector that could overexpress them by a ubiquitously active promoter CAG (consisting of the cytomegalovirus enhancer fused to the chicken beta-actin promoter) (Figure 4A).

Comparable amounts of wildtype Cx50 or P88L were expressed in GFP^+^ cells as determined by cellular immunofluorescence staining (Supplementary Figure S1). GFP^+^, EdU^+^, and GFP^+^EdU^+^ double-positive cells were counted after 24 and 48 h. There was no difference in the ratios of GFP^+^EdU^+^/GFP^+^ cells among groups (Supplementary Figure S2), implying that overexpression of *P88L* does not affect LECs proliferation, which probably explains why the patients do not have a phenotype of microphthalmia.

On the other hand, cell scratch wound assay showed that overexpression of human *GJA8* had a similar effect as vector (GFP), but overexpression of *P88L* caused a significantly slower rate of wound closure than those of wildtype and vector (Figures 4B,C), indicating that cells with P88L have a slower migration rate.

### P88L Inhibits Voltage Gating of Cx50 and Decreases the Membranal Ion Permeability of Human LECs

**FIGURE 5:**
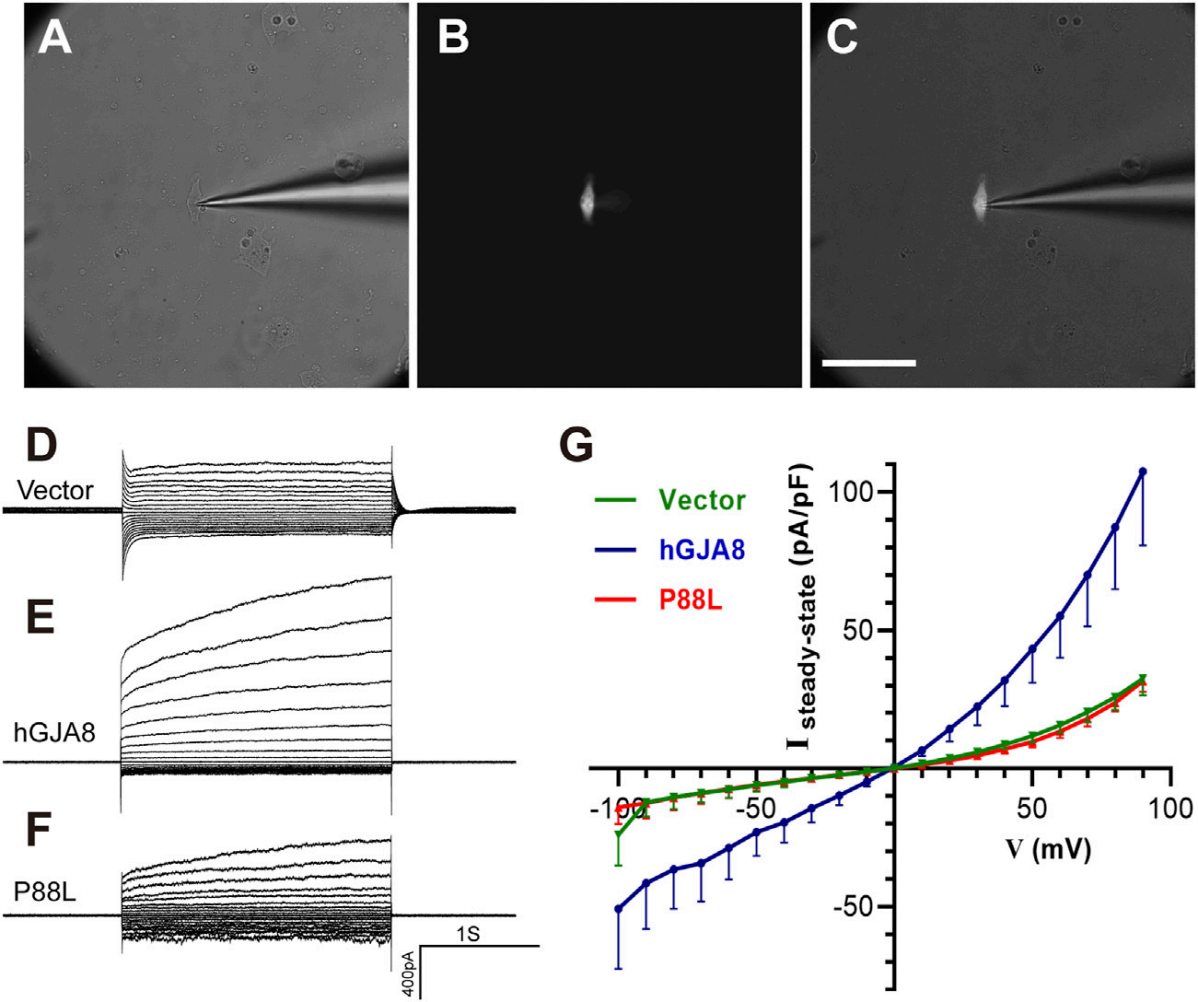
The P88L mutation inhibits hemichannel currents of Cx50 in human LECs. **(A–C)** Representative images show patch-clamp recordings of isolated GFP-positive cells under white light only **(A)**, blue light only **(B)**, and both white and blue lights **(C)**. Scale bar: 80 µm. **(D–F)** Steady-state currents from pulses were plotted as a function of membrane voltage. Representative voltage-dependent transmembrane current traces of LECs transfected with pCIG-vector **(D)**, *-hGJA8* **(E)** or -*P88L* **(F)** were measured at a holding potential of 0 mV and subjected to voltage pulses ranging from -100 to 90 mV in 10 mV steps. **(G)** Population steady-state current density–voltage relationships from human LECs expressing pCIG-vector, -hGJA8 or -P88L. Data is represented as mean ± SEM. Sample sizes: *N* = 12 for vector and P88L, *N* = 11 for hGJA8. *p* < 0.0001 for interaction and row factor, *p* = 0.0061 for column factor, with two-way ANOVA analysis.

To examine if P88L mutation compromises membranal voltage gating, human LECs were transfected with pCIG-vector, -*hGJA8* or -*P88L* mutant. To selectively evaluate the hemichannel conductance, isolated GFP^+^ cells were chosen (Figures 5A–C). Membrane currents were recorded while cells were clamped to different membrane voltages. We found that LECs expressing pCIG-vector, -hGJA8 or -P88L all displayed bigger currents at positive membrane voltages compared to the negative ones, particularly LECs with hGJA8 (1,263 ± 155 pA at 90 mV versus -546 ± 154 pA at −100 mV), indicating that Cx50 hemichannel is voltage-dependent. The LECs showed outward currents at positive potentials in a voltage-dependent manner (Figure 5D), consistent with low expression of GJA8 in these cells. Upon overexpression of GJA8, the outward currents were amplified significantly (107 ± 26 pA/pF versus 32 ± 6 pA/pF) (Figures 5E,G), and the inward current at negative membrane potential showed much lower than pCIG-vector (−51 ± 22 pA/pF versus −24 ± 11 pA/pF); however, both the inward and outward currents were dampened in cells expressing P88L (31 ± 4 pA/pF versus 107 ± 26 pA/pF) (Figures 5F,G). These results thus indicated that the P88L mutant protein inhibited the ionic currents in a voltage-dependent manner in human LECs.

## Discussion

There are four subclasses (GJA, GJB, GJC and GJD), around 20 members of connexins in human, sharing a topology of 4 transmembrane domains, 2 extracellular loops, and 3 intracellular regions (N-terminus, intracellular loop and C-terminus) (Beyer and Berthoud, 2018). Each hemichannel is assembled by the oligomerization of six homomeric or heteromeric subunits of Cxs, and two hemichannels dock end-to-end to form a gap junction channel. Hemichannels and channels allow the exchange of ions, metabolites and small molecules such as second messengers between a cell and its extracellular environment or between neighbor cells, respectively (Esseltine and Laird, 2016; García et al., 2016; Aasen et al., 2018; Jindal et al., 2021). However, how the differential tissue-specific expression of each connexin and the ratio of hemichannel-to-channel are controlled is not well understood.

There is no direct blood supply to mature lenses, and fiber cells depend on hemichannels to exchange ions and metabolites with extracellular aqueous humor while relying on gap junction channels for intercellular material swaps (Mathias et al., 2007; Mathias et al., 2010; Berthoud et al., 2020; Totland et al., 2020). Given that Cx50 is highly expressed in mature fiber cells and exists predominantly in the hemichannels rather than channels, we surmise that Cx50 may be the major player for exchanging ions and metabolites between the cells and the environmental humor (Mathias et al., 2010), and hence critical for maintaining lens homeostasis and physiology, which could be the primary reason to induce cataract when mutated.

Accumulating evidence from genetic sources and biochemical, cellular and physiological data indicates that the molecular mechanisms of connexin mutation-induced cataracts can attribute to four major causes, including disruption of lens development, impairment of lens transparency, alteration of channel and hemichannel electrical properties, disturbance of protein trafficking to trigger stress responses, or combinations of these causes (Mathias et al., 2010). The majority of the disease-causing mutations in Cx50 are dominant, indicating that these are either dominant-negative or gain-of-function mutations. The mutations are scattered across all the domains from C- to N-terminus (Figure 2C), demonstrating the integral requirement of all regions. One of the most frequently mutated regions in Cx50 is located between amino acid residues 40–60th, which directly influence the pore and channel and their mutations may result in significant perturbation to gating properties, conductance and free energy of the open-closed state (Myers et al., 2018).

The four transmembrane domains of Cx50 form the central channel and pore. The new mutation P88L is located in the second transmembrane domain. The P88 residue does not form the channel wall directly; however, it is highly conserved evolutionarily and most likely plays an essential role in the protein structure and function. A few mutations of the residue, P88T, P88S and P88Q, have been reported previously to cause various types of congenital cataracts (Shiels et al., 1998; Arora et al., 2006; Vanita et al., 2008a; Ge et al., 2014; Berry et al., 2020). Consistent with these reports, there was no microphthalmia or microcornea symptom associated with the P88L mutation, implying that P88 mutations probably do not perturb the LECs proliferation *in vivo*. Cell culture and other *in vitro* experiments showed that the membrane distribution of gap junction plaques and membrane-floating proteins were substantially different in the mutants, indicating a disruption of normal function by P88 mutations.

The mechanism of how Cx50 is involved in LECs proliferation and differentiation is unclear. Shi et al. showed that Cx50 C-terminus directly interacts with E3 ubiquitin ligase Skp2 and regulates lens cell-cycle progression and differentiation by modulating expression of Skp2 (Shi et al., 2015). However, there should be other mechanisms since this discovery cannot reconcile all the genetic findings. For example, a much higher ratio of microcornea/microphthalmia symptoms were associated with mutations in the first transmembrane domain and extracellular loop, which are known to participate in the structure and function of the central pore and channel. As another example, frameshift mutations causing loss of C-terminus or C-terminus point mutations have been found not to be associated with microcornea/microphthalmia. We speculate that intercellular material exchanges, such as cAMP and ions, may also be important for LECs proliferation and differentiation.

It is well known that precursor cell migration is crucial for the cell fate commitment and/or maturation during development. As an example, loss of Lhx1 causes mislocation of the differentiating migratory horizontal precursors and these displaced cells finally commit apoptosis (Poche et al., 2007). It is very complicated that how LECs migration and differentiation are regulated. Surface LECs at different positions are exposed to unique microenvironment defined by neighbor tissues such as cornea, iris and retina. As a result, the BMP, FGF, Notch, Wnt signaling molecules play crucial roles controlling LECs migration and differentiation (Bassnett and Šikić, 2017; Cvekl and Zhang, 2017; McAvoy et al., 2017). The *in vitro* data showed that P88L mutant deterred LECs migration to some extent that it is probably insufficient to interrupt LECs differentiation *in vivo*. However, proper cell migration is essential for the delicate assembly of lens structure as evidenced in the post-operative capsular opacification (PCO) (Raj et al., 2007).

Whole exome sequencing has greatly expedited the discovery of causative genetic mutations of Mendelian diseases, in which a high percentage of mutations occur in the exon regions. Targeted exome sequencing enables rapid identification of common and rare genetic variants at a much affordable cost. In China, its cost still continues to drop and it is being gradually adapted in more and more hospitals and institutes. We anticipate that it will soon become a common procedure in genetic counselling and clinical practice.

## Data Availability

The datasets presented in this study can be found in online repositories. The names of the repository/repositories and accession number(s) can be found below: https://ngdc.cncb.ac.cn/gsa-human/HRA001484.
