# Supplementary figures and images for: Identification of a New Mutation p.P88L in Connexin 50 Associated with Dominant Congenital Cataract
Source: Front Cell Dev Biol. 2022 Apr 21;10:794837. doi: 10.3389/fcell.2022.794837 (PMC9068895; doi:10.3389/fcell.2022.794837)

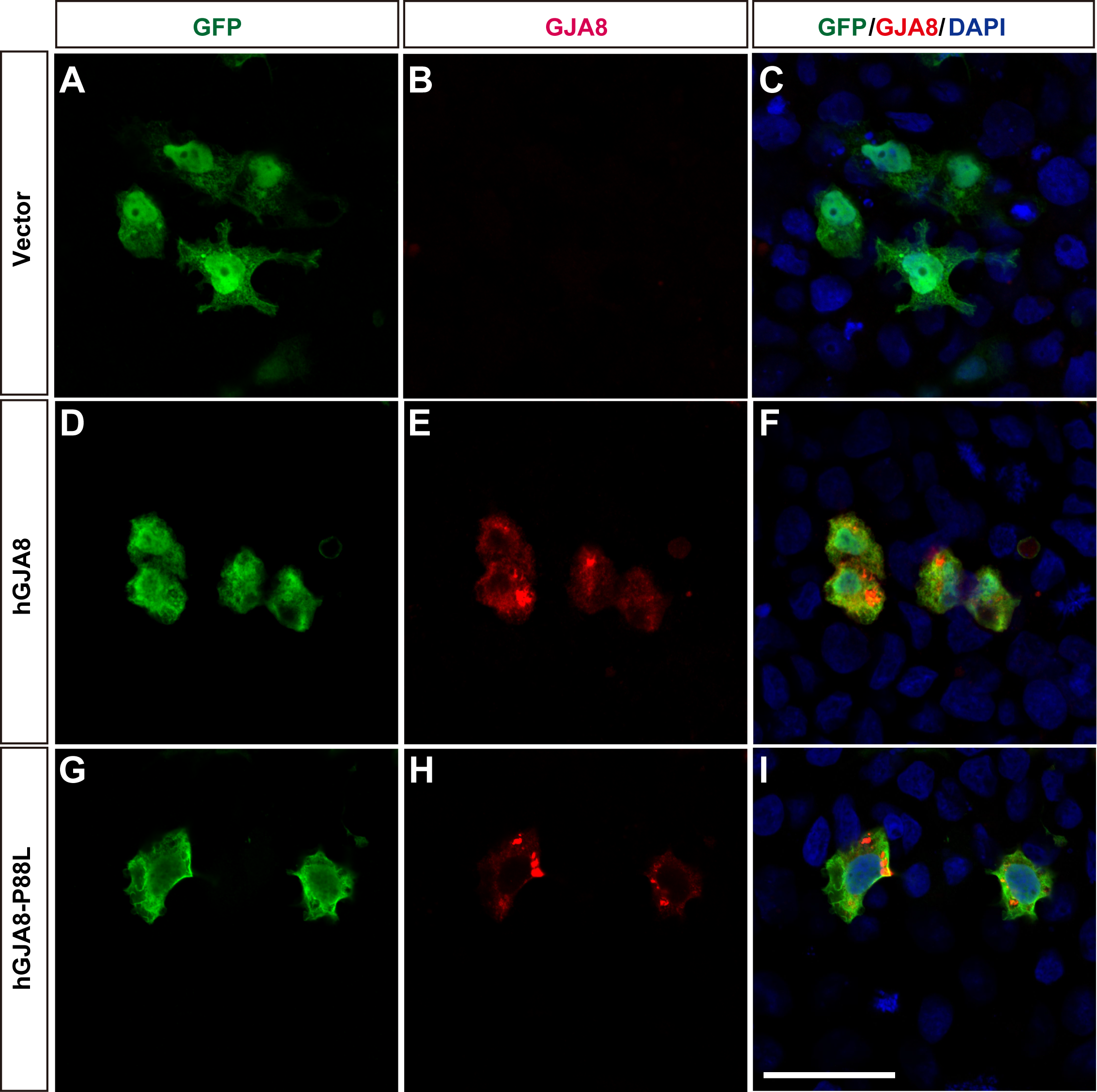

Supplement: Supplementary file 1 [file Figure6.TIF]

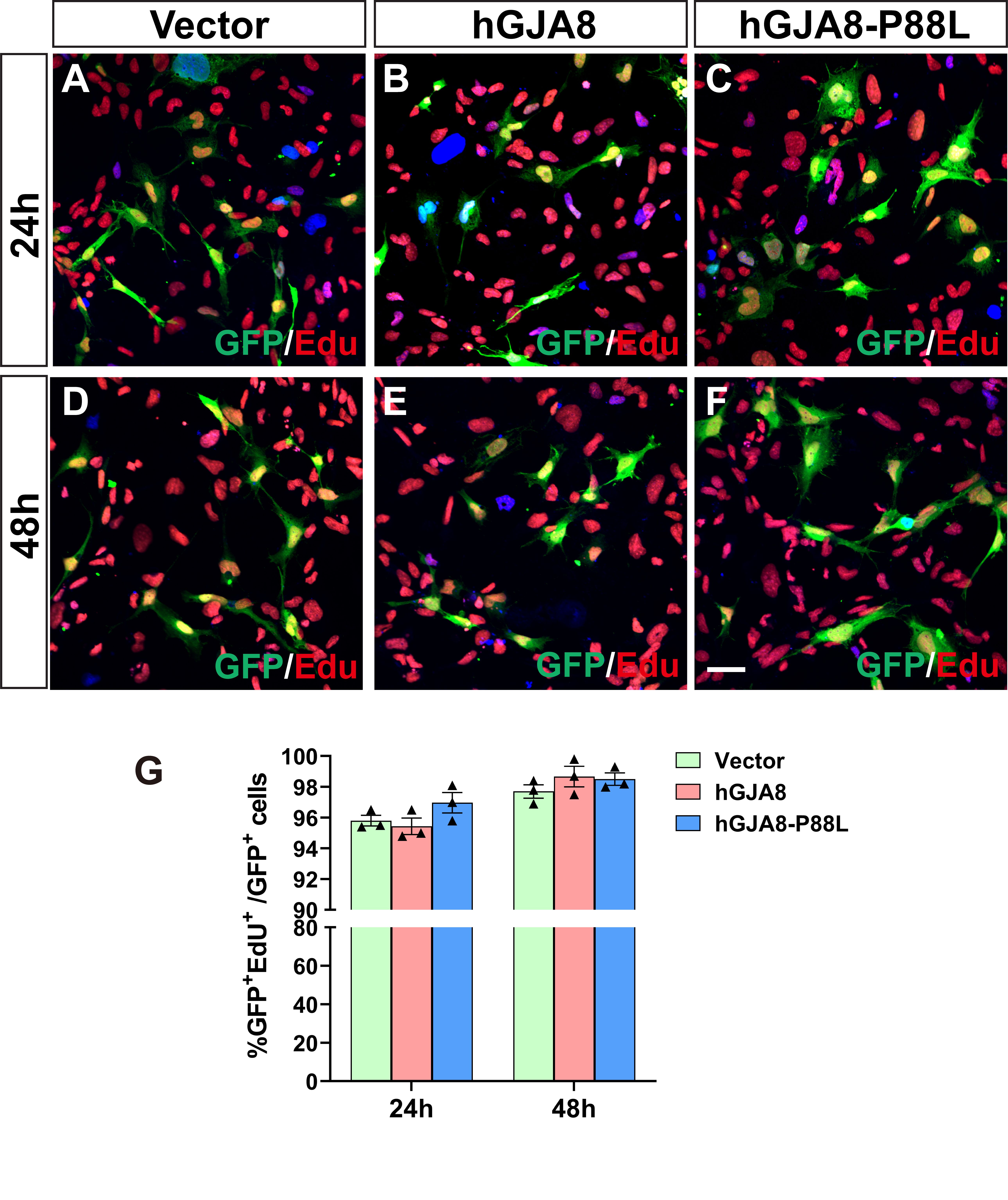

Supplement: Supplementary file 2 [file Figure7.JPEG]
